# Neue Aspekte in der Ätiologie und Therapie der jugendlichen Anorexia nervosa – ein postuliertes biopsychosoziales Modell und die Auswirkungen der COVID-19-Pandemie

**DOI:** 10.1007/s00103-024-03856-y

**Published:** 2024-03-18

**Authors:** Beate Herpertz-Dahlmann, Brigitte Dahmen, Ingar M. Zielinski-Gussen, Jochen Seitz

**Affiliations:** 1 Klinik für Psychiatrie, Psychosomatik und Psychotherapie des Kindes- und Jugendalters der RWTH Aachen, Neuenhofer Weg 21, 52074 Aachen, Deutschland; 2 Klinik f. Psychiatrie, Psychosomatik und Psychotherapie des Kindes- und Jugendalters, LVR-Klinikum Essen, 45147 Essen, Deutschland

**Keywords:** Anorexia nervosa, Umwelteinflüsse, Genetik, Darm-Gehirn-Achse, Home Treatment, Anorexia nervosa, Environmental influences, Genetics, Gut-brain axis, Home treatment

## Abstract

Die Anorexia nervosa ist eine der häufigsten chronischen Erkrankungen des Jugendalters mit einer hohen Mortalität. Ihre Behandlungsbedürftigkeit hat während der COVID-19-Pandemie vor allem im Kindes- und Jugendalter zugenommen. Der Artikel zeigt neue Forschungsergebnisse zur Ätiologie der Erkrankung auf, insbesondere zur genetischen Disposition und zu metabolischen Veränderungen. Vor dem Hintergrund der steigenden Behandlungszahlen während der COVID-19-Pandemie wird die Bedeutung der Gen-Umwelt-Interaktion diskutiert. Der zweite Schwerpunkt des Artikels bezieht sich auf neue Behandlungsmethoden. Neben dem experimentellen Einsatz biologischer Interventionen werden auch neue psychotherapeutische Behandlungsstrategien vorgestellt. Im Vergleich zur früheren Behandlung der Anorexia nervosa wird der intensiven Einbeziehung der Eltern in die Therapie eine hohe Bedeutung beigemessen. Dies zeigt sich insbesondere durch die Entwicklung der Behandlung zu Hause (Home Treatment). Die Konzeption der Anorexia nervosa als metabopsychiatrische Erkrankung ist mit der Hoffnung auf neue Forschungs- und Therapieansätze verbunden.

## Abstract (en)

Anorexia nervosa is one of the most frequent chronic disorders of adolescence associated with a high mortality. During the COVID-19-pandemic, the number of hospitalized children and adolescents with anorexia nervosa significantly increased. This article outlines new research findings to decode the etiology of this serious disorder, especially a genetic disposition and changes of metabolism. Against the background of increasing rates during the COVID-19 pandemic, the importance of the gene-environment interaction is discussed, and new treatment forms are described. Besides the development of new biological treatment strategies, there is also some important progress in psychotherapeutic interventions. Carers should always be integrated when treating children and adolescents with anorexia nervosa, which is especially emphasized in the new “home treatment” setting. The new concept of anorexia nervosa as a metabo-psychiatric disorder gives us hope for new research ideas and treatment strategies in this often-debilitating disorder of childhood and adolescence.

## Hintergrund

Die Anorexia nervosa (AN) gehört zu den häufigsten chronischen Erkrankungen des Jugendalters und ist nach den Suchterkrankungen die psychische Störung mit der höchsten Mortalität [1]. In den letzten Jahren hat eine intensive Forschung zur Ätiologie dieser Erkrankung, die bis Ende des letzten Jahrhunderts vornehmlich auf psychodynamischen Erklärungsmodellen beruhte, sowie zu wirksameren Behandlungsmodellen stattgefunden. Dieser Artikel hat sich zum Ziel gesetzt, neue Entwicklungen sowohl in Bezug auf Fragen zu Ursachen als auch zur Therapie darzustellen und den Einfluss der COVID-19-Pandemie auf die Entstehung der Störung und ihre Prävalenz zu verdeutlichen. Dabei wird vor allem der biologischen Ursachen- und Therapieforschung Raum gegeben. Der Anstieg der Prävalenz der Erkrankung während der Pandemie lässt die Frage nach der Bedeutung von Umwelteinflüssen stellen. Zusätzlich werden neue psychotherapeutische Behandlungsansätze sowohl in Bezug auf das Setting als auch auf die Einbeziehung der Bezugspersonen dargestellt, die aufgrund der Zunahme der Prävalenz im Jugendalter als auch aufgrund der hohen Mortalität und Chronifizierungsrate dieser Erkrankung dringend notwendig erscheinen.

Kernmerkmale der Erkrankung sind eine *ausgeprägte Gewichtsphobie*, eine meist *erhebliche Einschränkung der Energiezufuhr* mit daraus folgendem Gewichtsverlust sowie *eine Störung der Wahrnehmung der eigenen Figur*. Grundsätzlich wird zwischen dem restriktiven Typus (Gewichtsreduktion vornehmlich durch Fasten) und dem Binge/Purge-Typus (Heißhungerattacken und/oder Maßnahmen, einer Gewichtszunahme entgegenzusteuern, wie Erbrechen, Laxanzienabusus etc.) unterschieden. In beiden aktuellen Klassifikationssystemen Diagnostic and Statistial Manual of Mental Disorders (DSM)‑5 und International Classification of Diseases (ICD-)11 wurde die *Amenorrhö *als diagnostisches Kriterium weggelassen, um bestimmte Patientengruppen (z. B. Männer, Kinder, Frauen mit hormoneller Kontrazeption) einschließen zu können. Gleichfalls wurde in beiden Systemen auf die Unterstellung *willkürlicher Handlungen *der Patientinnen^1^ verzichtet (Wegfall der „Weigerung“, an Gewicht zuzunehmen, sowie der Krankheitsverleugnung). Diese Veränderung der Definition hat dazu beigetragen, dass die Eruierung biologischer Ursachen mit der Konsequenz entsprechender Therapieentwicklung Eingang in die Forschung finden konnte.

Neben der typischen AN gewinnt die *atypische Anorexia nervosa* (AAN) in jüngster Zeit an Bedeutung. Sie wird im DSM‑5 zu den sog. spezifischen Essstörungen und in der ICD-11 zu der Kategorie „Andere spezifische Anorexia nervosa“ gezählt. Die AAN erfüllt alle Kriterien der AN bis auf das Gewichtskriterium. Die körperlichen Folgen dieser Erkrankung sowie die psychische Komorbidität unterscheiden sich nicht von der der typischen AN; die Prognose scheint ebenfalls vergleichbar zu sein [2, 3].

In der jüngsten Zeit sind die AN und die AAN des Kindes- und Jugendalters sehr in den Blickpunkt gerückt, da ihre Prävalenz (und damit wahrscheinlich auch Inzidenz) während der COVID-19-Pandemie deutlich zugenommen hat. Dies wird im Folgenden im Abschnitt „Epidemiologie“ dargestellt. Der Einfluss der COVID-19-Pandemie auf die Häufigkeit der Erkrankung wird in einem eigenen Abschnitt dargestellt. Ziele dieses Artikels sind darüber hinaus die Darstellung tiefgreifender Veränderungen der Forschungsansätze zu Ursachen (s. Abschnitt „Ätiologie“ mit genetischen Ursachen und Erkenntnissen zur Darm-Hirn-Achse) als auch der (psycho)therapeutischen Ansätze. Dabei ist zu beachten, dass es sich hier um ausgewählte Aspekte handelt, die nicht für das gesamte Forschungsspektrum bei der AN repräsentativ sind.

## Epidemiologie (unter besonderer Berücksichtigung der COVID-19-Pandemie)

Die Erkrankung an AN ist nicht an ein bestimmtes Lebensalter gebunden; ein Erkrankungsgipfel liegt in der Adoleszenz. Bis zum 18. Lebensjahr sind ca. 50 % der Betroffenen erkrankt [4]. Über alle Altersgruppen hinweg tritt die AN bei ca. 4 % der Frauen und 0,3 % der Männer auf (Lebenszeitprävalenz; [5]); möglicherweise liegt die Prävalenz beim männlichen Geschlecht höher als in den meisten Studien angegeben. Da sich die diagnostischen Kriterien und die Messinstrumente vorwiegend auf Frauen beziehen, ist eine Unterschätzung der wahren Prävalenz nicht unwahrscheinlich. Die Punktprävalenz bei adoleszenten Mädchen liegt bei ca. 1 %. Während die Prävalenz der AN im Erwachsenenalter eher unverändert ist, hat sie im Jugendalter, vor allem aber im Kindesalter, in den letzten Jahren deutlich zugenommen. Besonders offensichtlich war dieser Anstieg während der COVID-19-Pandemie, auch wenn schon vorher eine Zunahme im Kindesalter beobachtet worden war [5, 6].

**Figure 1:**
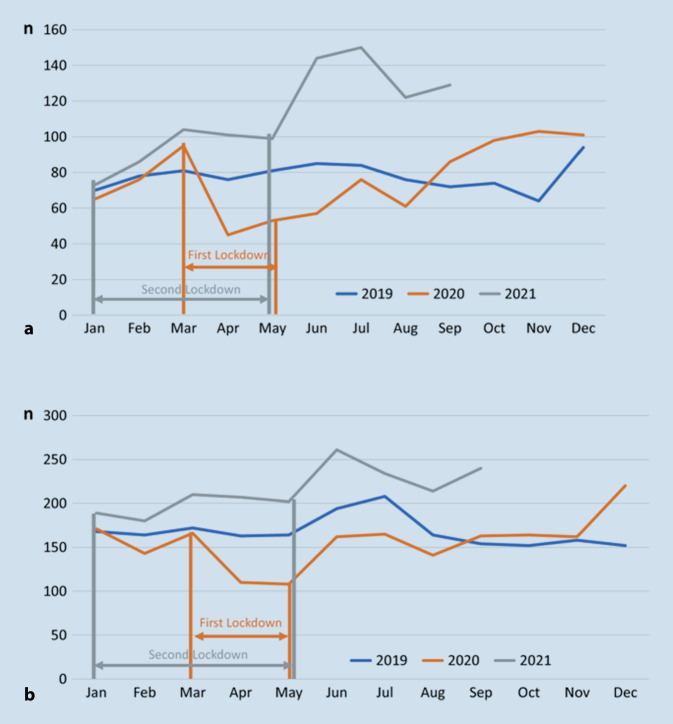

Bundesweite Erhebungen bei den gesetzlichen Krankenkassen weisen darauf hin, dass es nach einem Rückgang der stationären Aufnahmen während des ersten Lockdowns zu einem erheblichen Anstieg sowohl bei den Jugendlichen als auch vor allem bei den Kindern kam (Abb. 1). Die Häufung bei den Kindern fand sich sowohl bei Mädchen als auch bei Jungen, beim weiblichen Geschlecht allerdings deutlich stärker. Insgesamt betrug die Zunahme stationärer Aufnahmen bei den Kindern im Vergleich zu der Vor-COVID-19-Zeit im Jahr 2019 ca. 40 %, die bei den Jugendlichen ca. 38 % [7]. Erwähnenswert ist die relativ stärkere Zunahme von stationären Aufnahmen bei beiden Geschlechtern in pädiatrische Kliniken während der Pandemie im Vergleich zu kinder- und jugendpsychiatrischen Kliniken. Dies hängt möglicherweise damit zusammen, dass gerade in den jüngeren Altersgruppen häufiger eine somatische Differenzialdiagnose in Erwägung gezogen wurde. Die deutliche Zunahme an behandlungsbedürftiger AN im Kindes- und Jugendalter fand sich nicht nur in Deutschland, sondern auch in europäischen und nordamerikanischen Ländern [8].

## „Gen-Umwelt-Interaktion“ während der COVID-19-Pandemie

Befragte man die Kinder- und Jugendlichen nach der Ursache für ihre Problematik während der COVID-19-Pandemie, berichteten unsere Patientinnen und die in anderen Studien, dass sie ihre Alltagsstruktur verloren hätten, insbesondere während der Lockdowns. Sie beklagten unzureichende sportliche Aktivitäten bei grundsätzlich weniger Außenaktivitäten, was ihre Angst vor einer Gewichtszunahme triggerte. Durch den nicht mehr stattfindenden Schulbesuch hatten die Kinder und Adoleszenten mehr Freizeit und Langeweile; sie beschäftigten sich mehr mit den sozialen Medien und hier vor allem mit Webseiten über Work-outs und Diät [8, 9]. Insgesamt konnte eine intensivere Nutzung der digitalen Aktivitäten bei Kindern und Jugendlichen festgestellt werden [10]. Bestimmte Influencer, die Work-out- und Ernährungsanleitungen gaben, gewannen besonders an Einfluss.

Allerdings weisen neuere Zahlen der gesetzlichen Krankenkassen darauf hin, dass auch nach Ende der Restriktionen zur Eindämmung der Pandemie weiterhin höhere stationäre Aufnahmeraten als in der Vor-COVID-19-Zeit registriert werden, deren Ursache nicht klar ist. Möglich wäre eine allgemeine psychische Labilisierung von Kindern und Jugendlichen durch die Pandemie, die sich auch nach dem Ende der Restriktionen noch nicht normalisiert hat. Aus der COVID-19-Pandemie sind aber in jedem Fall wichtige Schlussfolgerungen für die Versorgung von Kindern und Jugendlichen zu ziehen: Zum einen muss die Frage der Isolierung junger Menschen bei einer allgemeinen Gesundheitsgefährdung sehr sorgfältig gestellt werden. Um Essstörungen – insbesondere AN – frühzeitig erkennen zu können, müssen regelmäßige ärztliche Vorsorgeuntersuchungen gewährleistet sein, ggf. durch mobile Teams. Nicht zuletzt sollte mit den Jugendlichen der Umgang mit sozialen Medien trainiert und auf mögliche Gefahren hingewiesen werden.

## Ätiologie

In den 70er-Jahren des letzten Jahrhunderts gelangte die Familie in den Blickpunkt der Ätiologie- und Therapieforschung psychischer Erkrankungen. Die Familie von Patientinnen mit AN wurde zum Prototyp der sog. psychosomatischen Familie [11], wobei den Patientinnen die Rolle des Problem- bzw. Symptomträgers der Familie zugeschrieben wurde. Eine besonders gravierende Konsequenz war die sog. Parentektomie, die bei einer stationären Behandlung einer betroffenen Tochter zu einer wochen-, teilweise monatelangen Trennung von Eltern und Kind führte. Noch heute berichten mir ehemalige mittlerweile erwachsene Patientinnen, dass es ihnen nicht möglich sei, mit ihren Eltern (meist der Mutter) über ihre damalige Erkrankung und Trennung zu sprechen, weil es für diese zu schambesetzt sei.

Die Erkenntnisse über den Einfluss des Hungerzustandes (Starvation) auf die Ausprägung der Symptomatik und zunehmendes Wissen über (neuro)biologische Zusammenhänge führten zu einer veränderten Sichtweise auf die Ätiologie der Erkrankung und zu einer Entlastung der Eltern.

Im Folgenden soll aktuelles Wissen zu genetischen Aspekten und metabolischen Veränderungen einschließlich neuer Erkenntnisse zu der Darm-Gehirn-Achse vermittelt werden, wobei es sich hier nur um Teilaspekte der neuen Erkenntnisse zur AN handelt.

### Genetische Disposition

**Figure 2:**
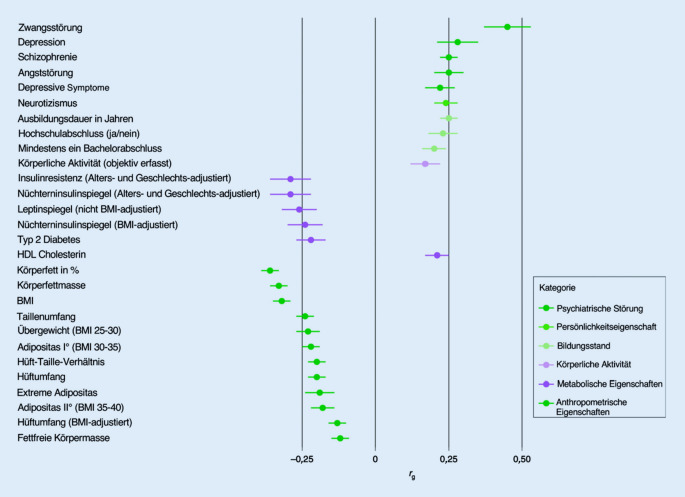

Schon lange wird ein familiär gehäuftes Auftreten von AN und anderen Essstörungen beobachtet. Weibliche Angehörige von Patientinnen mit AN haben ein ca. 10-fach erhöhtes Risiko, an einer Essstörung (nicht beschränkt auf AN) zu erkranken [12]. Studien bei ein- und zweieiigen Zwillingspaaren weisen auf einen Anteil der genetischen Varianz bei der AN von über 50 % hin, insbesondere wenn man sich an den klinisch-diagnostischen Kriterien orientiert [13, 14]. Die letzte Publikation einer genomweiten Assoziationsstudie (GWAS), die durch eine weltweite Probensammlung möglich wurde, erfolgte 2019 [15]. Die Gruppe um C. Bulik konnte insgesamt 8 signifikante chromosomale Regionen für die AN aufzeigen, die insgesamt 121 Gene umfassen, die vor allem im Gehirn exprimiert werden [14, 15]. Zusätzlich wurden genetische Korrelationen gefunden, die sich einerseits auf andere psychische Störungen, andererseits auf metabolische Veränderungen und das Körpergewicht beziehen (Abb. 2). Eine hohe positive genetische Korrelation besteht z. B. zu Zwangserkrankungen und depressiven Störungen, die Ärzten und Therapeuten als häufige komorbide Erkrankungen von AN sehr gut aus der klinischen Praxis bekannt sind. Weitere positive genetische Korrelationen fanden sich zu körperlicher Aktivität, zu einem hohen Bildungsniveau sowie zu einem akademischen Abschluss.

Sehr bedeutsam sind aber vor allem die Ergebnisse in Bezug auf negative genetische Korrelationen mit somatischen Parametern, insbesondere zu Fasteninsulin, Insulinresistenz und Leptin sowie zu Diabetes mellitus Typ 2. Viele Forscher sehen die AN mittlerweile als das metabolische Spiegelbild der Adipositas an, sodass diese Essstörung nicht mehr als ausschließlich psychische, sondern als metabopsychiatrische Störung definiert wird [15]. Ähnlich, wie es adipösen Menschen schwerfällt, ihr Gewicht nach einer Gewichtsreduktion auf einem niedrigeren Niveau zu stabilisieren, ergeht es auch Patientinnen mit AN. Es gelingt ihnen nicht, ein normales Gewicht beizubehalten.

Es ist aus Platzmangel nicht möglich, weitere – genetische und klinische Zusammenhänge – mit anderen somatischen oder psychischen Erkrankungen darzustellen, wie z. B. zum Diabetes mellitus Typ 1, Morbus Crohn, Zöliakie. Hierzu wird auf andere Quellen verwiesen ([16–18]; Abb. 2).

### Der Einfluss der Darm-Hirn-Achse

Im letzten Jahrzehnt konnte in Tierexperimenten gezeigt werden, dass das Füttern von Stuhlextrakt übergewichtiger Mäuse auf keimfrei aufgewachsene normalgewichtige Mäuse zu einer Gewichtszunahme der letzteren führte [19]; umgekehrt konnte die Transplantation von Stuhl, der von untergewichtigen Kindern mit Nahrungsmangelerkrankungen stammte, zu Untergewicht bei primär normalgewichtigen Mäusen führen [20]. Diese Beobachtungen unterstützten die Hypothese, dass die Zusammensetzung des Darmmikrobioms Einfluss auf die Gewichtsentwicklung hätte. Weitere Befunde führten zu zusätzlichen Erkenntnissen: In einer japanischen Studie wurde die Entwicklung der Nachkommen von Mäusen beobachtet, denen Stuhlextrakte von Patientinnen mit AN übertragen wurden. Die Nachkommen zeigten eine geringere Gewichtszunahme, einen geringeren Appetit und eine schlechtere Futterverwertung als Tiere, denen man den Stuhlextrakt von gesunden Kontrollpersonen transplantiert hatte [21]. In einer ähnlichen Untersuchung – allerdings nicht bei den Nachkommen, sondern als direkte Übertragung auf die Versuchstiere – konnte das Ergebnis nicht repliziert werden [22]. Eine rezente Studie konnte aber ein der Hata-Studie (2019; [21]) entsprechendes Ergebnis finden: Die Empfängertiere zeigten nach Stuhltransplantation von Patientinnen mit AN eine ausgeprägtere Gewichtsabnahme als die Kontrolltiere, wenn sie weniger Futter als üblich erhielten [23].

In einer eigenen Untersuchung mit immerhin 57 Patientinnen konnten wir zeigen, dass sich die Mikrobiomzusammensetzung im akuten Hungerzustand und vor allem bei den Patientinnen mit dem höchsten Untergewicht deutlich von dem gesunder gleichaltriger Kontrollpersonen unterscheidet. Mit zunehmendem Gewicht wurde die Diskrepanz zu dem Mikrobiom der Gesunden geringer; allerdings zeigten sich auch nach einem Jahr und nach Erreichen des Zielgewichtes noch Unterschiede zwischen den Patientinnen und den Kontrollen, sodass sich weiterhin die Frage stellt, ob die veränderte Mikrobiomzusammensetzung ein State- (akuter Krankheitszustand) oder Trait-Phänomen (dauerhafte Veränderung) der Patientinnen mit AN ist [24]. Unklar bleibt ebenfalls, ob ein verändertes Mikrobiom Ursache (z. B. bei einer genetischen Disposition für AN) oder Folge der Erkrankung (Epiphänomen bei verminderter Nahrungszufuhr und Nahrungszusammensetzung) ist. Es gibt Hypothesen, dass die COVID-19-Pandemie durch die Veränderung der Lebensgewohnheiten (veränderte Reisegewohnheiten, Desinfektionsmittelgebrauch, Ernährung, weniger Bewegung) ebenfalls das Mikrobiom beeinflusst haben könnte [25], wobei ein Rückschluss, dass dies zu einer veränderten Prävalenz der AN geführt habe, bei dem derzeit unzureichenden Wissensstand nicht gerechtfertigt wäre.

In jedem Fall könnten aber die o. g. Erkenntnisse Auswirkungen auf die Therapie der AN haben: So besteht die Hoffnung, dass durch eine Veränderung der Ernährung, Applikation von bestimmten Nahrungsergänzungsmitteln (z. B. Omega-3-Fettsäuren), lebenden Bakterien [26] oder durch eine Stuhltransplantation von Gesunden eine Veränderung des Mikrobioms bewirkt werden könnte, die zu einer Gewichtsnormalisierung oder -stabilisierung beitragen würde.

### Umwelteinflüsse

Da Umweltfaktoren für die Entstehung der AN gut bekannt sind und ihnen in der bisherigen Literatur ein hoher Stellenwert eingeräumt wurde, wird diesen hier aus Platzmangel weniger Raum gegeben, da vornehmlich neue Erkenntnisse dargestellt werden sollten.

Das westliche Schlankheitsideal spielt als auslösender Faktor eine bedeutsame Rolle bei der Entstehung der adoleszenten und kindlichen AN. Fast immer geht der AN eine Phase der Diät und der intendierten Gewichtsreduktion voraus. Besonders deutlich wurde dies während der COVID-19-Pandemie (s. oben).

Auch familiäre Faktoren und der Einfluss von Gleichaltrigen (vor allem eine hohe Bedeutung von sog. gesundem Essen, Gewicht, Sport, aber auch negative Kommentare bezüglich des Aussehens) können zu der Entstehung einer AN beitragen [27]. Auch Mobbing scheint ein Auslösefaktor für Essstörungssymptome zu sein [28], allerdings für die AN geringer als für andere Essstörungen [29]. In Bezug auf familiäre Faktoren ist es oft schwierig zu erkennen, welche bereits vor oder erst nach der Erkrankung relevant wurden (post oder propter; [30], s. auch Absatz unten „Belastung von Bezugspersonen“). Allerdings gibt es keinerlei wissenschaftliche Erkenntnisse, dass familiäre Ursachen die einzige oder primäre Ursache von Essstörungen sind [31].

Negative Lebensereignisse können ebenfalls eine Rolle spielen, aber in geringerem Ausmaß als bei den anderen Essstörungen wie der Bulimia nervosa und der Binge-Eating-Störung oder bei der Depression [32].

## Neue Therapiemethoden

### Biologisch-experimentelle Therapiemethoden

Neben der Manipulation des Darmmikrobioms wurde basierend auf der Kenntnis, dass die Konzentration von Leptin im Serum bei AN-bedingtem Untergewicht deutlich erniedrigt ist, in Fallstudien die Substitution von Leptin erprobt. Es zeigte sich die gewünschte Reduktion der körperlichen Aktivität, ein Rückgang der um das Essen kreisenden Gedanken, der inneren Unruhe, der Depression und der Gewichtsphobie. Weitere Studien, insbesondere nach Abschluss der Therapie und als Katamnese müssen folgen [33, 34], bevor eine endgültige Bewertung erfolgen kann.

Weitere bisher nur in Therapiestudien eingesetzte Verfahren sind die Neuromodulation [35] sowie seit Kurzem die psychodelische Substanz Psylocybin, von der man sich aufgrund der hohen Komorbidität der AN mit depressiven und Zwangserkrankungen einen Fortschritt verspricht ([36]; auch hierzu wird auf die einschlägige Literatur verwiesen).

### Home Treatment – ein neues psychotherapeutisches Setting

Therapieansätze aus den USA und Großbritannien machten deutlich, dass eine Einbeziehung der Eltern in die Behandlung den Therapieerfolg erheblich verbesserte. Die *familienbasierte Therapie (FBT*), die ursprünglich von Russell et al. [37] am Maudsley Hospital in London entwickelt wurde, ist eine primär ambulante Behandlung (die heute z. T. in stationäre Behandlungssettings integriert wird), bei der die Eltern wichtige co-therapeutische Funktionen übernehmen. So kontrollieren die Eltern z. B. in der ersten Behandlungsphase die Nahrungsaufnahme, das Ausmaß der körperlichen Bewegung und die Entwicklung des Gewichtes. In kontrollierten Studien hat sich die FBT im Vergleich zur Individualtherapie im Kurzzeitverlauf als wirksamer erwiesen [38], bei Langzeitverläufen ist eine höhere Effektivität weniger evident [39]. Leider ist die FBT in Deutschland kein Richtlinienverfahren und wird daher auch nicht von den gesetzlichen Krankenkassen finanziert.

Der Erfolg der Aktivierung der Eltern hatte großen Einfluss auf die Entwicklung und Etablierung neuer Therapieverfahren. So wurde an der Klinik für Psychiatrie, Psychosomatik und Psychotherapie des Kindes- und Jugendalters der RWTH Aachen in der letzten Dekade eine randomisiert-kontrollierte Studie zum Vergleich von tagesklinischer und vollstationärer Behandlung durchgeführt. Dabei zeigte sich ein Jahr nach Beginn der tagesklinischen Behandlung eine ähnlich positive Gewichts- und mentale Entwicklung wie nach der stationären Behandlung [40]. Eine weitere Folgeuntersuchung nach 2,5 Jahren ergab eine noch positivere Bilanz: Die tagesklinischen Patienten hatten ein signifikant höheres Gewicht und eine geringere stationäre Wiederaufnahmerate [41]. Leider wird in Deutschland eine tagesklinische Behandlung der AN immer noch nicht an allen kinder- u. jugendpsychiatrischen Kliniken durchgeführt, obwohl die Prognose der tagesklinisch behandelten Patienten wahrscheinlich besser ist. Deutschland hat mit über 100 Behandlungstagen eine der längsten stationären Aufnahmedauern der westlichen Welt, wobei der Heilungserfolg nicht größer als bei kürzeren stationären Aufenthalten ist [42, 43].

Trotz der im Vergleich zur stationären Behandlung geringeren Wiederaufnahmerate nach tagesklinischer Behandlung war diese aus unserer Sicht mit ca. 30 % der Patientinnen nach 2,5 Jahren immer noch zu hoch. Viele Familien berichteten von Konflikten in der Essenssituation. Eltern und Patientinnen wünschten sich eine intensivere und professionelle Unterstützung zu Hause.

Seit den 1980er-Jahren wurden meist in England Home-Treatment-Programme zur Behandlung kinder- und jugendpsychiatrischer Störungen entwickelt. Diese dienten einerseits der Versorgung von Kindern und Jugendlichen in akuten psychischen Krisen [44, 45], andererseits dem Übergang von stationärer in ambulante Behandlung zur Verkürzung stationärer Aufenthalte und zur Stabilisierung des Erreichten [46]. Letztere dienten als Modelle zur Entwicklung der „Zu-Hause-Behandlung“ (Home Treatment) bei Jugendlichen mit AN.

Da es sich bei der AN um eine ggf. letale Erkrankung handelt, wurde eine Pilotstudie durchgeführt, um die Machbarkeit der Behandlung und eine mögliche Gefährdung der Patienten zu eruieren. In der Pilotstudie konnte im Rahmen des Home Treatment die Zunahme auf ein gesundes Körpergewicht erreicht werden, das auch noch ein Jahr nach der Aufnahme stabil war. Patientinnen und Eltern waren mit der Behandlung sehr zufrieden [47], und die Motivation der Patientinnen, ihr Gewicht zu normalisieren und die Krankheit zu überwinden, verbesserte sich im Verlauf der Behandlung und war ein Jahr nach stationärer Aufnahme am größten [48].

Aufgrund der optimistischen Ergebnisse der Pilotstudie wurde mit Unterstützung des Innovationsfonds des Gemeinsamen Bundesausschusses eine randomisiert-kontrollierte Studie initiiert. In dieser Studie mit insgesamt 5 Kliniken wird der Behandlungserfolg des Home Treatment (HoT) mit dem der üblichen Behandlung (stationär oder tagesklinisch) verglichen. Die Durchführung der Studie setzt allerdings eine ausführliche Schulung der beteiligten Mitarbeiter voraus.

Bei den Teilnehmerinnen handelt es sich grundsätzlich um sehr kranke Patientinnen, die einer stationären Behandlung bedürfen. Zur somatischen und psychologischen Stabilisierung werden die Patienten zuerst stationär aufgenommen und für 6–8 Wochen auf der Station behandelt. Die stationäre Behandlungszeit wird genutzt, um die Patientinnen und ihre Familien optimal auf die Behandlung zu Hause vorzubereiten. In maximal 8 Wochen erfolgt die Stabilisierung der somatischen Parameter, und die Patientin muss lernen, selbstständig zu essen. Sind diese Bedingungen erfüllt, kann die Patientin in das HoT aufgenommen werden, im anderen Fall erfolgt eine Weiterbehandlung auf der Station, im Verlauf meist auch in der Tagesklinik.

**Figure 3:**
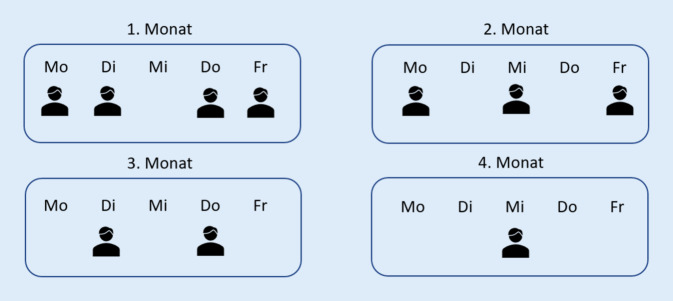

Da die Rückfallgefahr erfahrungsgemäß in der Zeit direkt nach Entlassung am größten ist [49], wurde das HoT-Programm in Bezug auf seine Intensität angepasst, d. h., in den ersten 4 Wochen nach Entlassung erfolgen die meisten, im letzten Monat die wenigsten Hausbesuche (Abb. 3).

An den Besuchen zu Hause sind mehrere Berufsgruppen beteiligt: Dazu gehören Ärzte und psychologische Psychotherapeuten, Ökotrophologen, Mitarbeiter der Pflege und Ergotherapeuten. Alle Mitarbeiter haben Erfahrung in der Behandlung von Essstörungen. Im Gegensatz zur stationsäquivalenten Behandlung (StäB) muss bei HoT keine wöchentliche fachärztliche Visite zu Hause erfolgen; dies gilt insbesondere in einer fortgeschrittenen Behandlungsphase bei Patientinnen mit AN, wenn medizinische Probleme oder Fragen nicht mehr im Vordergrund stehen. Allerdings kann im Notfall immer eine ärztliche Konsultation angefordert werden oder in der Klinik erfolgen. Überdies ist das HoT-Team während der üblichen Arbeitszeiten immer erreichbar; außerhalb der Arbeitszeiten nimmt der Dienstarzt/die Dienstärztin diese Aufgabe wahr.

Ein besonderes Kennzeichen der HoT (auch vielfach im Gegensatz zu StäB) ist, dass dieselben Therapeuten, die die Patientin während des stationären Aufenthaltes betreut haben, die Behandlung zu Hause fortsetzen. Das hat die Einführung der Behandlung erheblich erleichtert, da das therapeutische Team der Familie bereits bekannt ist und es dadurch vielen Familien leichter fällt, den Behandlern „die Tür zu öffnen“.

## Die Belastung von Bezugspersonen

Das Risiko für die Entwicklung einer psychischen Störung bei den Bezugspersonen ist um das *Doppelte* bis *Vierfache* im Vergleich zur Normalbevölkerung erhöht. Ein Viertel der Angehörigen leidet unter Angststörungen, 30 % an depressiven und mehr als ein Drittel an ausgeprägten Stresssymptomen [50]. Viele Eltern leiden selbst an einer Essstörung. Die Belastung von Angehörigen essgestörter Patienten ist höher als die von Patienten mit Schizophrenie oder Depression [51]. Vergleichende Untersuchungen haben aufgezeigt, dass die *Betreuungszeit* für Angehörige von Patienteninnen mit AN mit 24 h/Woche signifikant höher ist als die für Angehörige von Patienten mit anderen psychischen oder somatischen Erkrankungen (Karzinome, Psychosen und Demenz) mit ca. 14 h/Woche [52, 53].

Die Behandlung der Patientinnen im häuslichen Umfeld erleichtert sowohl das Erkennen psychischer Probleme bei den Angehörigen als auch deren Unterstützung. In unserer Pilotstudie konnte ein deutlicher Rückgang der depressiven Symptomatik bei den Müttern der im Home Treatment behandelten Patientinnen nachgewiesen werden.

Wir erhoffen uns vom Home Treatment eine geringere Rückfallrate und weniger stationäre Wiederaufnahmen sowie eine Verbesserung der allgemeinen psychischen Gesundheit der Patientinnen und ihrer Angehörigen. Sollte sich diese Behandlung als effektiv erweisen, könnte sie ggf. in die Routinebehandlung übernommen werden.

## Fazit

In den letzten Jahren hat eine intensive Forschung zur Ätiologie, Diagnostik und Behandlung der AN stattgefunden und zu neuen Behandlungsmethoden motiviert. In Bezug auf die Ätiologie der Erkrankung spielen genetische, metabolische, psychologische und soziale Risikofaktoren eine bedeutende Rolle. Allerdings ist es bei dem derzeitigen Kenntnisstand nicht möglich, eine Einordnung des Stellenwertes der jeweiligen Faktoren vorzunehmen, der aus unserer Sicht individuell unterschiedlich sein kann. So werden z. B. bei einigen Patientinnen mit hoher familiärer Belastung genetische Ursachen, aber auch erlerntes Essverhalten eine wichtige Rolle spielen, bei anderen hat eine traumatische Erfahrung in der Kindheit eine entscheidende Bedeutung. Vor dem Hintergrund der Hypothese zur *Gen-Umwelt-Interaktion* (s. Abschnitt Genetik) könnte postuliert werden, dass die durch die COVID-19-Pandemie bedingte sehr belastende Umweltsituation zu einer Zunahme der krankheitsauslösenden Faktoren, insbesondere von Diätmaßnahmen oder auch zu Stimmungsverschlechterungen, geführt hat, die ihrerseits bei genetisch vulnerablen Kindern und Jugendlichen eine Erkrankung an AN zur Folge hatte, die in weniger belasteten Zeiten möglicherweise nicht erkrankt wären.

Die zukünftige Therapie dieser metabolisch-psychischen Erkrankung wird mit hoher Wahrscheinlichkeit nicht mehr nur auf psychotherapeutischen Verfahren beruhen, sondern auch biologisch-medizinische Maßnahmen umfassen. Darüber hinaus sollte sie eine intensive Einbindung der Familie ermöglichen. Es ist zu hoffen, dass diese neuen Behandlungschancen die Prognose dieser immer noch lebensgefährlichen und zur Chronifizierung neigenden Erkrankung verbessern und den jungen Patientinnen ein gesundes Leben ermöglichen.

## Footnotes

1 Da die Mehrzahl der an AN erkrankten Personen weiblich ist, wird hier der Begriff „Patientin“ gewählt. Damit sind aber immer auch männliche Patienten gemeint.
